# Protein N-terminal acetylation is required for embryogenesis in Arabidopsis

**DOI:** 10.1093/jxb/erw257

**Published:** 2016-07-06

**Authors:** Jinlin Feng, Ruiqi Li, Junya Yu, Shuangshuang Ma, Chunyan Wu, Yan Li, Ying Cao, Ligeng Ma

**Affiliations:** College of Life Sciences, Capital Normal University, and Key Laboratory of Plant Gene Resources and Biotechnology for Carbon Reduction and Environmental Improvement, Beijing Municipal Government; Beijing 100048, China

**Keywords:** Arabidopsis, asymmetric division, embryogenesis, hypophysis, NatA, protein N-terminal acetylation.

## Abstract

Early embryonic development generates precursors of all major cell types in Arabidopsis. Among these precursors, the hypophysis divides asymmetrically to form the progenitors of the quiescent center and columella stem cells. A great deal has been learnt about the mechanisms that control the asymmetric division of the hypophysis and embryogenesis at the transcriptional level; however, no evidence of regulation at the co- or post-translational level has been reported. Here, we show that mutation of the catalytic subunit (Naa10) or auxiliary subunit (Naa15) of NatA, an N-terminal acetyltransferase that catalyzes protein N-terminal acetylation, produces an embryo-lethal phenotype. In addition, Naa10 and Naa15 were found to interact physically *in planta*. Further analysis revealed that the observed embryonic patterning defects started at the early globular stage and that the asymmetric division of the hypophysis was irregular; thus, no quiescent center progenitor cells were generated in *naa10* and *naa15* embryos. We further observed that the polar distributions of auxin and its efflux carrier PIN1 were disturbed in *naa10* embryos. Our results suggest that NatA is required for asymmetric division of the hypophysis and early embryonic patterning in Arabidopsis, and provides a link between protein N-terminal acetylation and embryogenesis in plants.

## Highlight

NatA, an N-terminal acetyltransferase, is required for asymmetric division of the hypophysis and early embryonic patterning in Arabidopsis, and provides a link between protein N-terminal acetylation and embryogenesis in plants.

## Introduction

Embryogenesis initiates higher plant development. Following fertilization, the zygote elongates and divides asymmetrically into a large basal cell and a small apical cell. The basal cell and its daughter cells continue to expand longitudinally and divide transversely to produce a file of cells. The uppermost daughter of the basal cell is the hypophysis, which generates the progenitors of the quiescent center (QC) and columella stem cells through asymmetric division at the globular stage (Scheres *et al.*, 1994; ten Hove *et al.*, 2015). Meanwhile, the apical cell generates the entire embryo, except for the QC and columella stem cells, by following a predictable sequence of cell divisions (Jenik *et al.*, 2007; Lau *et al.*, 2012). Cell fate during embryogenesis is determined at the globular stage; thereafter, in Arabidopsis development proceeds according to an internal genetic program (Jenik *et al.*, 2007; ten Hove *et al.*, 2015).

The specification of the hypophysis is regulated by auxin; thus, mutations affecting auxin responses, perception, transport, or biogenesis can cause defects in the division of the hypophysis and formation of the root meristem (Robert *et al.*, 2015; Smit and Weijers, 2015). An auxin response maximum exists in the hypophysis; it is generated by polar localization of the auxin efflux transporter PIN1 and polar auxin transport (Friml *et al.*, 2003). This response maximum triggers changes in gene expression that control the specification of the hypophysis. There are at least three transcriptional regulatory pathways involved in auxin-mediated gene expression and hypophysis specification in Arabidopsis: (1) an auxin-responsive transcription factor, MP/AFR5, interacts with BDL/IAA12 to directly target *TOM7*, a bHLH transcription factor gene (Hardtke and Berleth, 1998; Hamann *et al.*, 2002; Schlereth *et al.*, 2010); (2) AFR9, another auxin-responsive transcription factor, works antagonistically with IAA10 to mediate the response to auxin and specification of the hypophysis (Rademacher *et al.*, 2012); and (3) TPL, a transcriptional corepressor, targets *PLT* family genes (Aida *et al.*, 2004; Long *et al.*, 2006; Galinha *et al.*, 2007). These pathways are integrated and co-operate to control hypophysis specification. In addition, homeobox transcription factor WOX family genes are involved in the control of hypophysis specification and early embryonic patterning in Arabidopsis (Haecker *et al.*, 2004; Breuninger *et al.*, 2008). Thus, much is known about the mechanisms underlying hypophysis specification and early embryonic patterning at the transcriptional level. However, little is known about its regulation at the co- or post-translational level.

N-terminal acetylation is one of the most common co-translational modifications in eukaryotes; about 50% of the proteins in yeast, 70–80% of the proteins in flies, and 80–90% of the proteins in humans and Arabidopsis undergo N-terminal acetylation (Silva and Martinho, 2015). N-terminal acetylation is catalyzed by a group of conserved N-terminal acetyltransferases (NATs). There are six distinct NATs in eukaryotes (NatA to NatF). Some NATs are protein complexes that require different catalytic and auxiliary subunits (e.g., NatA, NatB, NatC, and NatE), while others are able to catalyze protein N-terminal acetylation independently of protein partners (NatD and NatF) (Starheim *et al.*, 2012). Protein N-terminal acetylation is involved in multiple cellular functions, including the regulation of protein–protein interactions, the control of protein stability by targeting certain proteins to the cell membrane for degradation, and the inhibition of protein targeting to the secretory pathway (Arnesen, 2011). Thus, protein N-terminal acetylation is a major determinant of the lifespan of various cellular proteins.

Pesaresi *et al.* (2003) reported that NatC is required for the biosynthesis of photosystem II core proteins in plants and for maintaining the abundance of thylakoid multiprotein complexes. Ferrandez-Ayela *et al.* (2013) demonstrated that mutations in the auxiliary subunit of NatB caused pleiotropic developmental defects, including defects in flowering-time regulation and defects in leaf, inflorescence, flower, fruit, and embryo development. Xu *et al.* (2015) revealed that a Nod-like receptor, SNC1, undergoes N-terminal acetylation by both NatA and NatB at its first and second methionine residues, respectively. NatA-mediated acetylation is a degradation signal, while NatB-mediated acetylation functions to stabilize SNC1 (Xu *et al.*, 2015). Thus, protein N-terminal acetylation is required for plant immune responses. Linster *et al.* (2015) verified that drought stress decreased the level of N-terminal-acetylated proteins, that abscisic acid down-regulated the abundance of NatA, and that decreases in NatA and N-terminal-acetylated protein abundance were necessary for drought tolerance in Arabidopsis. It was also reported that leaf-type FERREDOXIN-NADP^+^ OXIDOREDUCTASE is N-terminally acetylated in Arabidopsis; however, the function of this modification is not understood (Lehtimäki *et al.*, 2014). These results suggest that protein N-terminal acetylation plays a critical role in plant development and survival.

In the present study, we found that mutations of Naa10 and Naa15, the catalytic and auxiliary subunits of NatA, respectively, led to disturbances in polarized auxin distribution and/or the auxin response maximum, and PIN1 subcellular localization in Arabidopsis embryos, resulting in defects in early embryonic patterning, including defects in the asymmetric division of the hypophysis and root meristem formation. Thus, this work demonstrates the critical role of NatA in embryonic patterning and provides a link between protein N-terminal acetylation and embryogenesis in plants.

## Materials and methods

### Plant materials and growth conditions

All plant materials used in this study were in the Columbia-0 ecotype (Col-0) background of *Arabidopsis thaliana*. The three *A. thaliana* mutants, *naa10-1* (CS850295), *naa15-1* (CS836292), and *naa15-3* (CS24056), were obtained from the Arabidopsis Biological Resource Center (ABRC; Columbus, OH, USA)). The T-DNA insertion sites in the three mutants were confirmed by PCR and sequencing. The primers used for PCR are given in Supplementary Table S1 at *JXB* online. Plant lines carrying *DR5::GFP* and *pPIN1::PIN1-GFP* were also obtained from the ABRC. Seeds were sterilized and placed on Murashige and Skoog medium containing 0.3% agar and 1% sucrose. After stratiﬁcation in the dark at 4 °C for 3 d, the plates were transferred to a Percival CU36L5 growth chamber (Percival Scientific, Perry, IA, USA) and held at 22 °C for 16h in the light and at 18 °C for 8h in the dark. After 8 d of growth, the plants were transferred to soil and cultivated in a greenhouse using the same photoperiod and temperature conditions.

Plant lines carrying different markers were crossed with *naa10-1* ^*+/−*^, and the progeny were identified by PCR and observed for GFP fluorescence under a fluorescence microscope. Those *naa10-1* ^*+/−*^ mutants carrying a homozygous GFP fluorescence marker were used in our subsequent experiments.

### Complementation of the *naa10* and *naa15* mutations

The *Naa10* coding sequence (CDS) fused to the 7xMyc tag driven by the 35S promoter was cloned into the binary vector pCambia1300. The resulting 35S::*Naa10*-Myc plasmid was introduced into *naa10-1* ^*+/−*^ by *Agrobacterium tumefaciens*-mediated transformation (Clough and Bent, 1998). T1 plants were selected using hygromycin and Basta plates, and then genotyped by PCR. The *Naa15* CDS fused to 3xFlag driven by its native promoter [1400bp upstream of the 5′-untranslated region (UTR)] was cloned into pCambia1300. The resulting *pNaa15*::*Naa15*-Flag plasmid was introduced into *naa15-3* ^*+/−*^ by *A. tumefaciens*-mediated transformation. T1 plants were selected on hygromycin and Basta plates, and then genotyped by PCR. The primers used for cloning the CDS of *Naa10* and *Naa15* are included in Supplementary Table S1.

### Ovule clearing and embryo observation

Fresh embryos were cleared for visualization using Hoyer’s solution [chloral hydrate:glycerol:water, 8:1:3 (w/v/v)] for minutes to hours depending on the developmental stage of each embryo (Berleth and Jürgens, 1993). Next, the cleared ovules with embryos were examined by differential interference contrast microscopy under a Zeiss Image 2 fluorescence microscope (Oberkochen, Germany). Confocal laser scanning microscopy (Zeiss LSM 5 Live confocal line-scanning microscope) was used to detect the fluorescent signals of the molecular markers. The excitation light source was set at 488nm for the detection of the GFP fluorescent signal, and 405nm for the detection of the DAPI fluorescent signal. Fresh embryos were isolated from ovules, mounted in 6% glycerol, and then observed by confocal microscopy.

### Yeast two-hybrid interaction assay

All of the genes tested were cloned into two vectors (pGADT7 and pGBKT7) and cotransformed into yeast strain AH109. The primers used for cloning the CDS of *Naa10* and *Naa15* are included in Supplementary Table S1. Transformation, yeast growth, and protein extraction were performed as described in the Clontech Yeast Protocols Handbook.

### Preparation of anti-Naa15 antibodies

The truncated protein corresponding to amino acid residues 610–872 of Arabidopsis Naa15 was used as the antigen to immunize rabbits to generate specific antibodies against Naa15.

### Immunoprecipitation

In *Nicotiana benthamiana*, total protein from leaves inoculated with agrobacterial strains containing either 35S::*Naa10*-Myc or 35S::*Naa15*-Flag, as well as 35S::*Naa10*-Myc and 35S::*Naa15*-Flag, were extracted with Native Extraction Buffer 1 (Liu *et al.*, 2010) and immunoprecipitated with anti-Myc or -Flag antibodies (Sigma, Madison, WI, USA). The immunoprecipitates were then separated by sodium dodecyl sulfate-polyacrylamide gel electrophoresis (SDS-PAGE) and immunoblotted with anti-Flag or -Myc antibodies.

In *A. thaliana*, total protein from wild-type or complemented *naa10-1* ^*–/–*^ transgenic plants was extracted using IP buffer (100mM Tris-HCl, pH 7.5, 150mM NaCl, 0.5% NP-40, 1mM PMSF, and a protease inhibitor cocktail), and then immunoprecipitated with anti-Myc or -Naa15 antibodies. The immunoprecipitates were separated by SDS-PAGE and immunoblotted with anti-Naa15 or -Myc antibodies.

## Results

### Naa10 is required for embryogenesis in Arabidopsis

To elucidate the role of protein N-terminal acetylation in plants, we investigated the function of NatA in Arabidopsis. A preliminary analysis revealed that Naa10, the catalytic subunit of NatA, is evolutionarily conserved from yeasts to humans and plants (see Supplementary Fig. S1).

**Fig. 1.**
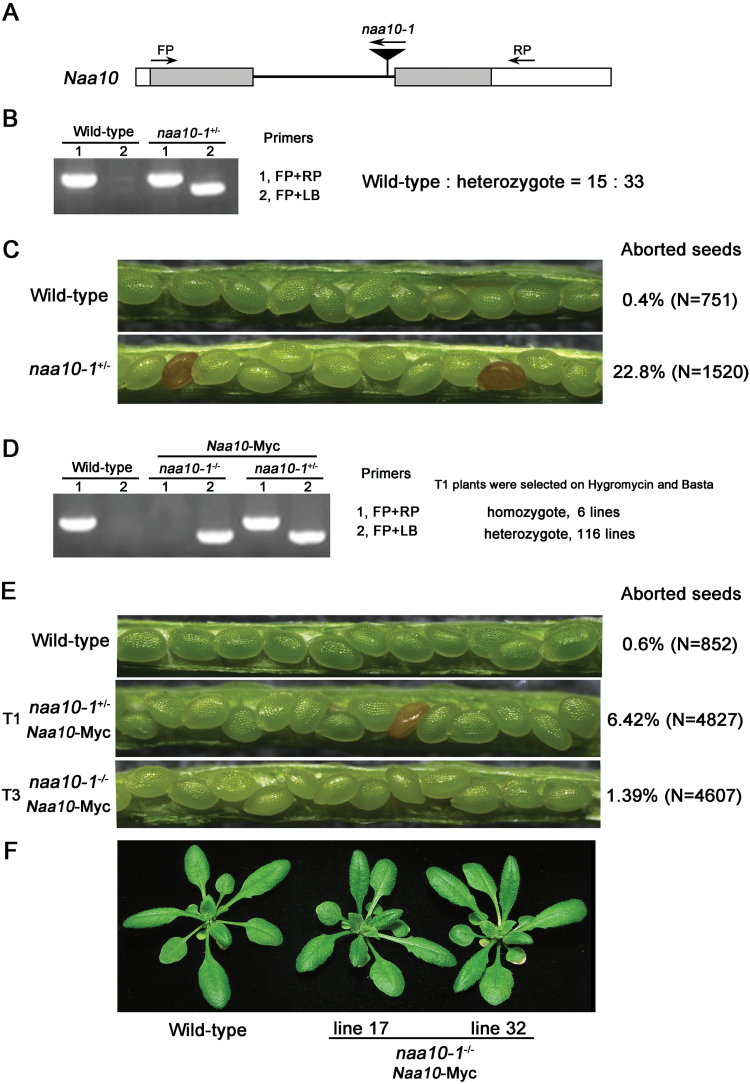
The mutation of Naa10 produced an embryo-lethal phenotype. (A) Schematic diagram of the T-DNA insertion in Arabidopsis *Naa10*. The mutant, *naa10-1* (CS850295), had a T-DNA insertion in intron 1. Grey boxes indicate exons, a black line indicates the intron, white boxes indicate UTRs, and arrowheads indicate the positions of the primers used for genotyping. (B) Genotyping of *naa10-1* by PCR. The ratio of wild-type to heterozygous *naa10-1* plants is shown. (C) Seed development in wild-type and *naa10-1* ^*+/–*^. The percentages of aborted seeds in wild-type and heterozygous *naa10-1* siliques are shown. (D) Genotyping of complemented *naa10-1* ^*+/–*^ transgenic plants. The *Naa10* CDS fused to the 7xMyc tag driven by the 35S promoter was cloned into pCambia1300. The resulting 35S::*Naa10*-Myc plasmid was introduced into *naa10-1* ^*+/–*^ by *A. tumefaciens*-mediated transformation. T1 plants were selected using hygromycin and Basta, and then genotyped by PCR as in (B). (E) Seed development in wild-type plants and *naa10-1* ^*+/–*^ plants complemented with *Naa10*-Myc at the T1 and T3 generations. The ratios of aborted seeds from plants with different genetic backgrounds are shown. (F) Twenty-five-day-old wild-type plants and *naa10-1* ^*-/–*^plants complemented with *Naa10*-Myc at the T1 generation. Lines 17 and 32 are independent, representative *naa10-1* ^*–/–*^-complemented lines.

To determine the role of Naa10 in Arabidopsis development, we obtained a T-DNA insertion mutant of Naa10 from the ABRC. Insertion of the T-DNA into the first intron of *Naa10* was confirmed by sequencing (Fig. 1A). No homozygotes were isolated from the progeny of a heterozygous *naa10* plant, and the ratio of wild-type to heterozygous plants was about 1:2 (Fig. 1B), indicating that the mutation was lethal in the homozygous state. In support of this prediction, about one-quarter of the seeds were aborted in the siliques of *naa10* heterozygous plants (Fig. 1C).

To verify that the embryonic phenotype observed in *naa10* was due to the defect in *Naa10*, we transferred Myc-tagged Naa10 (Naa10-Myc) into heterozygous *naa10* plants. Among 122 independent transgenic lines, homozygous *naa10* plants were identified in six lines at the T1 generation (*χ* ^2^=6.15, *P*<0.05) (Fig. 1D). In addition, the embryonic defect observed in *naa10* was complemented by the transformation of Naa10 into *naa10* plants (*χ* ^2^=519.34, *P*<0.01 for *naa10* ^*+/–*^ and *naa10* ^*+/–*^ *Naa10-MYC* transgenic plants; *χ* ^2^=3.71, *P*>0.05 for wild-type and *naa10* ^*+/*-^ *Naa10-MYC* transgenic plants) (Fig. 1E), and adult homozygous *naa10* plants harboring Naa10-Myc were indistinguishable from wild-type plants (Fig. 1F). These results suggest that Naa10 is required for embryogenesis in Arabidopsis.

### Naa10 is required for asymmetric division of the hypophysis and formation of the QC in the root meristem

**Fig. 2.**
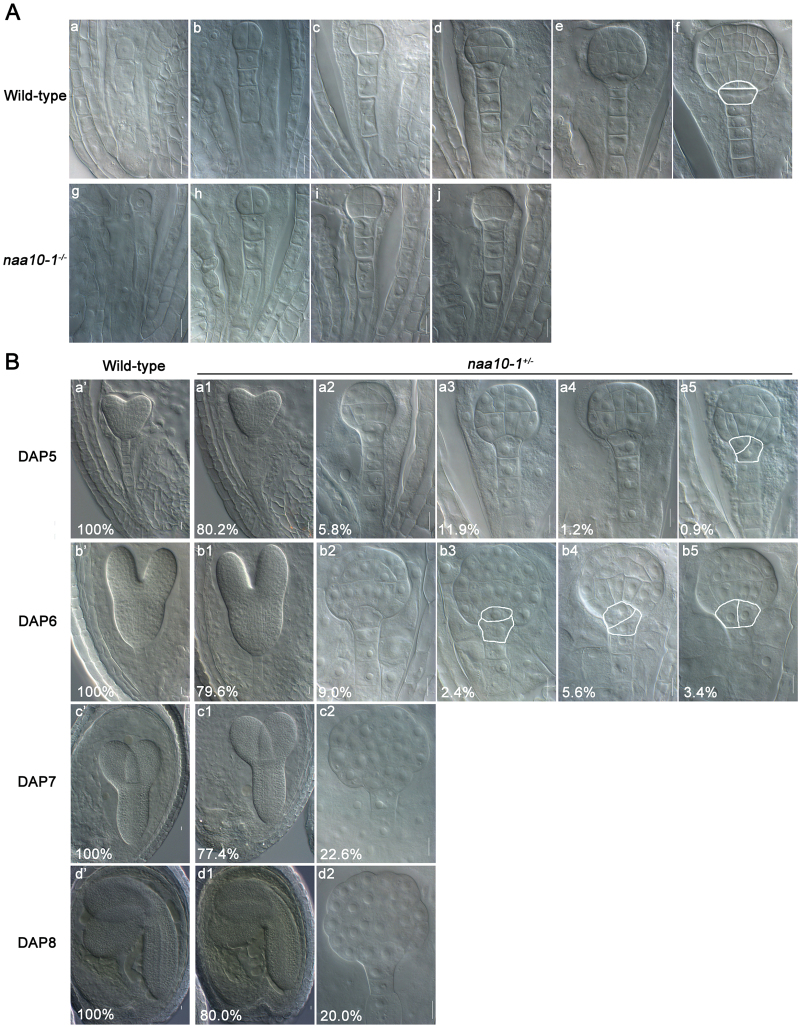
Naa10 is required for asymmetric division of the hypophysis and formation of the QC in the root meristem during embryogenesis in Arabidopsis. (A) Embryogenesis in wild-type (a–f) and *naa10-1* ^*+/–*^ (g–j) plants was examined by differential interference contrast microscopy. No morphological changes between wild-type and *naa10-1* ^*–/–*^ were observed before the dermatogen stage (d and j). Horizontal, asymmetric division of the hypophysis is marked with a white line in (f). (a, g) 1-cell stage; (b, h) 2/4-cell stage; (c, i) octant stage; (d, j) dermatogen stage; (e) early globular stage; (f) late globular stage. Scale bars =10 μm. (B) Embryogenesis in wild-type (a’–d’) and *naa10-1* ^*+/–*^ (all other images) plants examined under differential interference contrast microscopy. Flowers of both wild-type and *naa10-1* ^*+/–*^ plants were pollinated with self-pollen; fixed at 2, 3, 4, 5, 6, 7, and 8 DAP; cleared; and observed under differential interference contrast microscopy. The proportions of *naa10-1* ^*+/–*^ embryos at different stages are shown here and in Table 1. (a2) Dermatogen stage; (a3) between the dermatogen stage and early globular stage; (a4, b2) early globular stage; (c2, d2) sunflower-like globular stage embryos. Abnormal division of the hypophysis is marked with a white line in a5 (askew division of the hypophysis), b3 (distorted lens-shaped cell), b4 (askew division of the hypophysis), and b5 (vertical division of the hypophysis). Scale bars =10 μm.

Embryogenesis starts with the division of the zygote, and it proceeds through several developmental stages to produce a mature embryo. To determine the developmental stage and developmental process mediated by Naa10, we analyzed embryonic development in wild-type and *naa10* plants in detail. The embryonic development of wild-type and *naa10* plants was indistinguishable from the one-cell stage to the dermatogen stage (Fig. 2A; Table 1). The earliest stage at which a defect was observed in *naa10* was the early globular stage (Fig. 2B; Table 1). When 100% of the wild-type embryos were at the heart stage [5 d after pollination (DAP)], about 20% of the embryos in the siliques of heterozygous *naa10* plants were still in the early or late globular stage (Fig. 2B; Table 1). Furthermore, about 20% of the embryos remained at the globular stage after all of the embryos in the wild-type plants and 80% of the embryos in the siliques of the heterozygous *naa10* plants had reached maturity (Fig. 2B; Table 1). These data indicate that Naa10 is required for embryogenesis beginning at the early globular stage.

**Table 1.** Developmental progression of embryogenesis in wild-type, *naa10-1* ^+/–^, and *naa15-3* ^+/–^ plants

DAP	1-Cell	2/4-Cell	Octant	Dermatogen	Early globular	Late globular	Heart stage	Torpedo embryo	Late torpedo embryo	Walking-stick stage	No. of seeds examined
Wild-type plants											
2	1.9%	93.3%	4.8%	–	–	–	–	–	–	–	210
3	–	0.7%	1.0%	52.8%	45.5%	–	–	–	–	–	290
4	–	–	–	–	8.2%	91.8%	–	–	–	–	329
5	–	–	–	–	–	–	100%	–	–	–	349
6	–	–	–	–	–	–	–	100%	–	–	356
7	–	–	–	–	–	–	–	–	100%	–	356
8	–	–	–	–	–	–	–	–	–	100%	360
*naa10-1* ^+/–^plants											
2	5.7%	87.0%	7.3%	–	–	–	–	–	–	–	228
3	–	4.5%	17.3%	53.5%	24.7%	–	–	–	–	–	404
4	–	–	2.8%	17.5%	14.7%	64.9%	–	–	–	–	285
5	–	–	–	–	19.8%	–	80.2%	–	–	–	328
6	–	–	–	–	–	20.4%	–	79.6%	–	–	377
7	–	–	–	–	–	22.6%	–	–	77.4%	–	368
8	–	–	–	–	–	20.0%	–	–	–	80.0%	365
*naa15-3* ^+/-^ plants											
2	6.3%	89.3%	4.4%	–	–	–	–	–	–	–	272
3	–	6.5%	13.8%	61.3%	18.4%	–	–	–	–	–	385
4	–	–	2.3%	12.3%	6.8%	78.5%	–	–	–	–	219
5	–	–	–	–	17.9%	–	82.1%	–	–	–	336
6	–	–	–	–	–	18.3%	–	81.7%	–	–	404
7	–	–	–	–	–	16.0%	–	–	84.0%	–	326
8	–	–	–	–	–	14.9%	–	–	–	85.1%	322

Root formation is initiated at the globular stage by the transverse, asymmetric division of the hypophysis. This division generates a large basal daughter cell and a small lens-shaped apical daughter cell, which is the progenitor of the QC. Given that the defect in embryogenesis in *naa10* emerged at the globular stage (Fig. 2A, B), we examined whether the development of the hypophysis was affected in *naa10*. Asymmetric division of the hypophysis occurred normally in wild-type plants (Fig. 2A), whereas it was irregular in *naa10* plants; no functional QC progenitor was created in *naa10* embryos (Fig. 2B). Therefore, Naa10 is required for asymmetric division of the hypophysis and formation of the QC in the root meristem.

### The auxin distribution pattern and PIN1 localization were disrupted in *naa10* embryos during embryogenesis

**Fig. 3.**
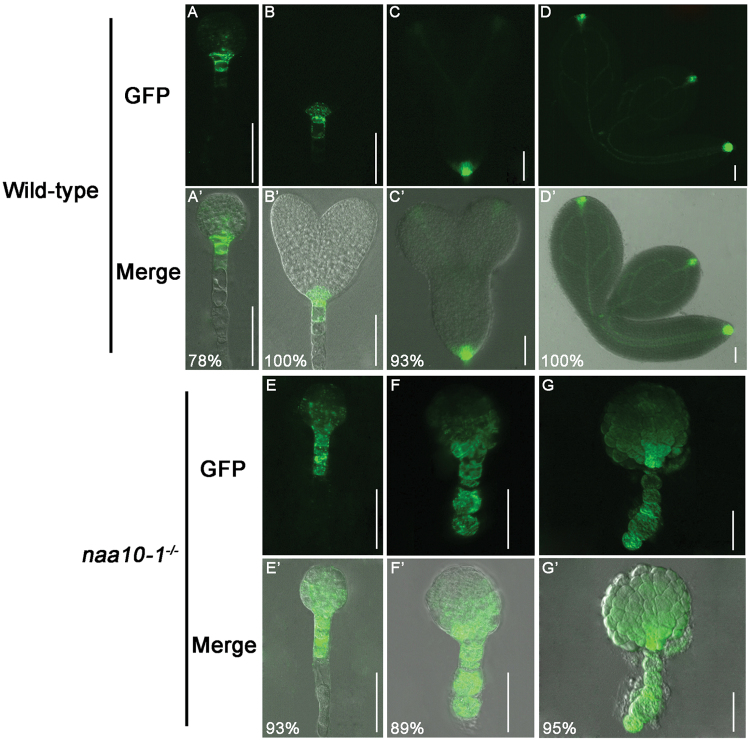
Naa10 is required for local auxin accumulation in the early embryonic stage during embryogenesis. (A–D) and (A’–D’) wild-type embryos; (E–G) and (E’–G’) *naa10-1* ^*–/–*^ embryos. (A–G) GFP signal; (A’–G’) merged images. DR5 was expressed in the root pole of the wild-type globular stage (A, A’) and heart stage (B, B’) embryos. DR5 was also expressed in wild-type cotyledon tips (C, C’) and in the vasculature of mature wild-type embryos (D, D’). DR5 was expressed throughout the arrested globular embryo as well as the suspensor in *naa10-1* ^*–/–*^ embryos (E–G). (B) and (E), (C) and (F), and (D) and (G) are embryos at the same time after fertilization, respectively. The number of embryos examined was 18, 13, 15, 10, 28, 27 and 22 for (A) to (G), respectively. Scale bars =50 μm.

Auxin has a profound effect on embryogenesis: the asymmetric distribution of auxin affects both apical–basal and radial patterning. The production of the lens-shaped QC progenitor cell is contingent on the establishment of an apical-to-basal auxin flux and the presence of an embryonic axis at the early globular stage. To determine the pattern of auxin distribution or auxin response in wild-type and *naa100* embryos, we first introduced a DR5-GFP reporter into *naa10* plants by crossing. Auxin was asymmetrically distributed in the uppermost cells of the suspensor, the hypophysis, and its daughter cells in globular wild-type embryos (Fig. 3), whereas the distribution or/and response of auxin in *naa10* embryos at the globular stage was nearly uniform (Fig. 3), indicating that the distribution of auxin or/and response maximum was disrupted in *naa10* plants during embryogenesis.

**Fig. 4.**
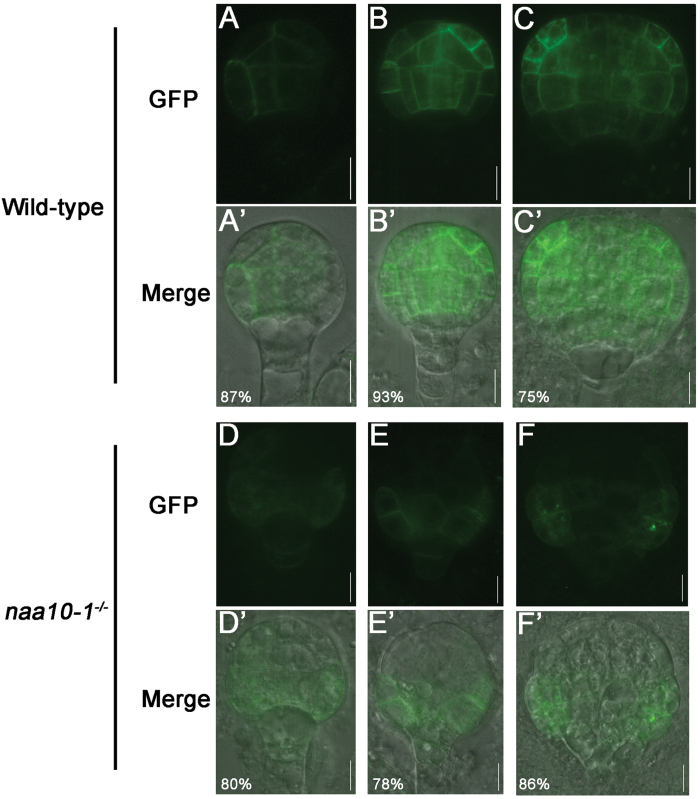
Naa10 is required for the polarized localization of PIN1 during early embryogenesis. (A–C) and (A’–C’) wild-type embryos; (D–F) and (D’–F’), *naa10-1* ^*–/–*^ embryos. (A–F), GFP signal; (A’–F’), merged images. PIN1 was present on all inner membranes of the proembryo until the dermatogen stage (A, A’); it became localized toward the basal end of the inner cells at the globular stage (B, B’). During the globular stage, PIN1 localization became polarized toward the flanks of the proembryo apex (C, C’). PIN1 was present throughout the basal half of the arrested *naa10-1* ^*–/–*^ globular embryos; this was assumed to be the hypophysis and suspensor (D–F and D’–F’). The number of embryos examined was 15, 15, 16, 20, 27 and 22 for (A) to (F), respectively. Scale bars =10 μm.

The asymmetric distribution of auxin in developing embryos is controlled by auxin transport. At the early globular stage, apical-to-basal transport is established by the asymmetric distribution of PIN auxin efflux carriers, and it persists throughout the plant life cycle (Steinmann *et al.*, 1999; Friml *et al.*, 2003). To understand why the asymmetric distribution of auxin was disrupted, we examined the distribution of PIN1 in *naa10* embryos. To this end, we transformed PIN1-GFP into *naa10* plants by crossing. PIN1 was preferentially localized to the basal membrane in the inner cells of wild-type embryos at the proembryo stage (Fig. 4); this is required for the transport of auxin to the hypophysis and QC. In contrast, the asymmetric localization of PIN1 to the inner cells of the proembryo was missing in *naa10* embryos (Fig. 4). Thus, Naa10 may be required for the polar localization of PIN1 to the inner cells of the proembryo and for the asymmetric distribution of auxin in embryos.

### Mutation of *Naa15* produced a similar embryonic phenotype to *naa10*

**Fig. 5.**
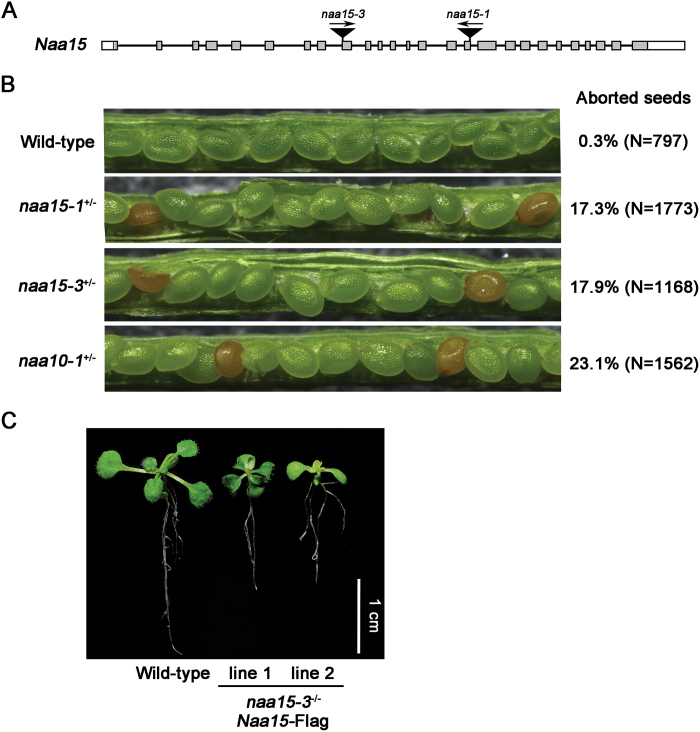
The mutation of Naa15 produced an embryo-lethal phenotype. (A) Schematic diagram of the two T-DNA insertions in Arabidopsis *Naa15*. The T-DNA insertions in *naa15-1* (CS836292) and *naa15-3* (CS24056) were in exons 16 and 9, respectively. Grey boxes indicate exons, black lines indicate introns, white boxes indicate UTRs. (B) Seed development in wild-type, *naa15-1* ^*+/–*^, *naa15-3* ^*+/–*^, and *naa10-1* ^*+/–*^ plants. The ratios of aborted to normal seeds in siliques from wild-type, *naa15-1* ^*+/–*^, *naa15-3* ^*+/–*^, and *naa10-1* ^*+/–*^ plants are shown. (C) Fifteen-day-old wild-type plants and *naa15-3* ^*–/–*^ plants complemented with *Naa15*-Flag at the T1 generation. The *Naa15* CDS fused to 3xFlag driven by its native promoter was cloned into pCambia1300. The resulting *pNaa15*::*Naa15*-Flag plasmid was introduced into *naa15-3* ^*+/–*^ by *A. tumefaciens*-mediated transformation. T1 plants were selected using hygromycin and Basta, and then genotyped by PCR. Lines 1 and 2 are independent *naa15-3* ^*–/–*^-complemented lines.

Some NATs are protein complexes that utilize different catalytic and auxiliary subunits. Naa10 and Naa15 are the catalytic and auxiliary subunits of NatA, respectively, in yeast and animal cells (Starheim *et al.*, 2012). We found that Naa15 is an evolutionary conserved protein from yeasts to humans and plants (see Supplementary Fig. S2). To examine the function of Naa15 in Arabidopsis, we obtained two *naa15* alleles, *naa15-1* and *naa15-3*, from the ABRC. The T-DNA insertion site for each allele was confirmed by sequencing (Fig. 5A). As before, no homozygous plants were isolated from the progeny of a heterozygous *naa15* plant, and aborted seeds were observed in the siliques of the two *naa15* alleles (Fig. 5B). In addition, the seed-aborted phenotype observed in *naa15* was complemented by the transformation of Flag-tagged Naa15 (Naa15-Flag) into *naa15* plants, and a homozygote of *naa15* was isolated from the transgenic lines (Fig. 5C). These results indicate that, similar to Naa10, Naa15 is required for embryogenesis in Arabidopsis.

**Fig. 6.**
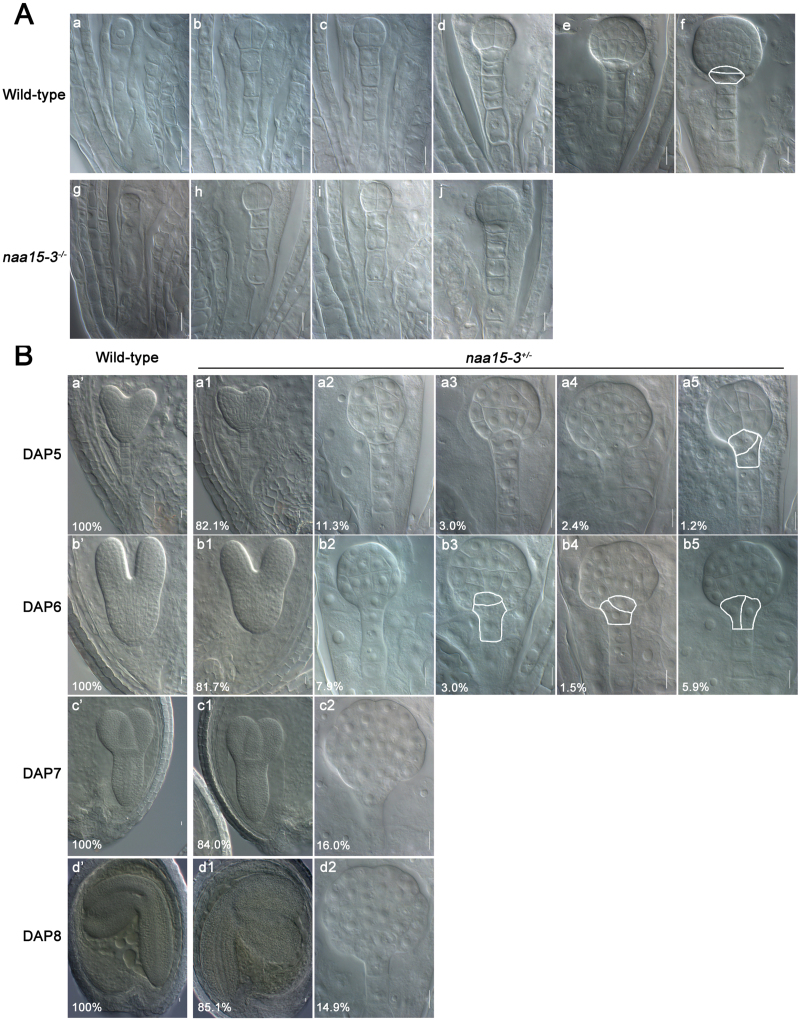
Naa15 is required for asymmetric division of the hypophysis and formation of the QC in the root meristem during embryogenesis in *Arabidopsis*. (A) Embryogenesis in wild-type (a–f) and *naa15-3* ^*+/*-^ (g–j) plants examined under differential interference contrast microscopy. No morphological changes between the wild-type and *naa15-3* ^*–/–*^ embryos were observed until the dermatogen stage (d, j). Horizontal, asymmetric division of the hypophysis is marked with a white line in (f). (a, g) 1-cell stage; (b, h) 2/4-cell stage; (c, i) octant stage; (d, j) dermatogen stage; (e) early globular stage; (f) late globular stage. Scale bars =10 μm. (B) Embryogenesis in wild-type (a’–d’) and *naa15-3* ^*+/–*^ (all other images) plants was examined under differential interference contrast microscopy. Flowers of both *naa15-3* ^*+/–*^ and wild-type plants were pollinated with self-pollen; fixed at 2, 3, 4, 5, 6, 7, and 8 DAP; cleared; and then observed under differential interference contrast microscopy. The proportions of *naa15-3* ^*+/–*^ embryos at different stages are shown here and in Table 1. (a2) Dermatogen stage; (a3) stage between the dermatogen and early globular stages; (a4, b2) early globular stage; (c2, d2) sunflower-like globular stage embryos. Abnormal division of the hypophysis in *naa15-3* ^*–/–*^ is marked with a white line in a5 (askew division of the hypophysis), b3 (distorted lens-shaped cell), b4 (askew division of the hypophysis), and b5 (vertical division of the hypophysis). Scale bars =10 μm.

Also similar to *naa10*, the defect in *naa15* was first observed at the early globular stage, and *naa15* embryos were still at the globular stage when the embryos of *Naa15* plants reached maturity (Fig. 6A, B; Table 1). Further, irregular cell division was observed in the hypophysis of *naa15* embryos, meaning that no functional QC progenitor was generated (Fig. 6B). Together, our results suggest that both Naa10 and Naa15 are required for embryogenesis in Arabidopsis.

### Mutation of Naa10 or Naa15 causes a defect in male transmission efficiency

In our analysis of *naa10* and *naa15* plants, we observed that the percentages of aborted seeds and abnormal embryos were less than 25% (Figs 1, 2, 5, and 6; Table 1). If the only function of these genes was in embryogenesis, the percentages of aborted seeds and abnormal embryos would be 25%. This result therefore appears to indicate the existence of defects in the gametocytes of both *naa10* and *naa15* plants. To examine this prediction, we measured the transmission efficiencies of male and female gametocytes from *naa10* or *naa15* plants in a reciprocal outcross between wild-type and heterozygous *naa10* or *naa15* plants. No defect in the transmission efficiency of female gametocytes was detected for *naa10* and *naa15*, whereas a decrease in the transmission efficiency of male gametocytes was detected for *naa10* and *naa15* (Table 2). Consistent with this result, the gametocyte transmission efficiency was also decreased in self-crossed heterozygous *naa10* or *naa15* plants (Table 2). Therefore, Naa10 and Naa15 are required for both embryogenesis and male gametocyte development in Arabidopsis.

**Table 2.** Segregation of *naa10* and *naa15* among the progeny of self-crossed and reciprocally outcrossed plants

Recipient × donor	No. of progeny	Antibiotic resistance		Transmission efficiency
		Susceptible^1^	Resistant^2^	
*naa10* ^+/–^ × *Naa10* ^+/+^	731	373 (51.03%)	358 (48.97%)	95.98%
*Naa10* ^+/+^ × *naa10* ^+/–^	570	371 (65.09%)	199 (34.91%)	53.64%
*naa15* ^+/–^ × *Naa15* ^+/+^	425	216 (50.82%)	209 (49.18%)	96.76%
*Naa15* ^+/+^ × *naa15* ^+/–^	584	395 (67.64%)	189 (32.36%)	47.85%
**Self-crosses**	**No. of progeny**	**Antibiotic resistance**		**Ratio of Bar^1^ to Bar^2^**
		**Bar^s^**	**Bar^r^**	
*naa10* ^+/–^ × *naa10* ^+/–^	2211	845 (38.22%)	1366 (61.78%)	1:1.62
*naa15* ^+/–^ × *naa15* ^+/–^	2952	1245 (42.17%)	1707 (57.83%)	1:1.37

^1^, susceptible to Basta, ^2^, resistant to Basta.

### Naa10 and Naa15 are in the same protein complex

**Fig. 7.**
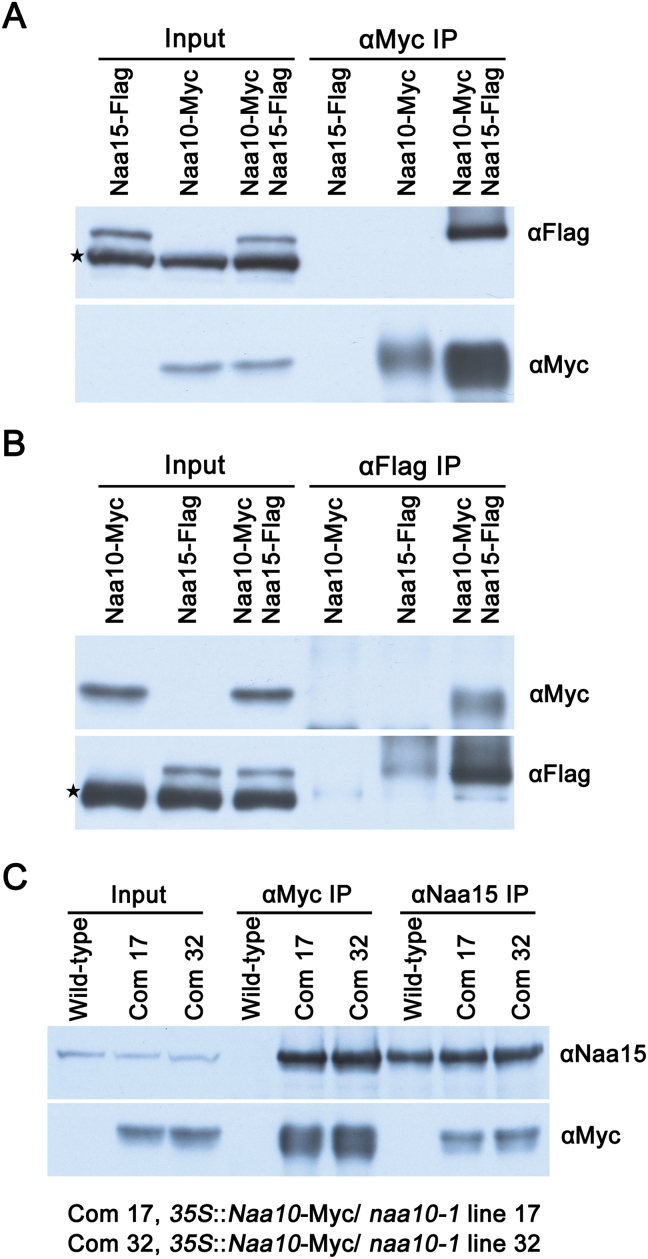
*In vivo* physical interaction between Naa10 and Naa15 in *N. benthamiana* and Arabidopsis. (A, B) Total proteins from plants inoculated with agrobacterial strains containing either 35S::*Naa10*-Myc or 35S::*Naa15*-Flag, as well as 35S::*Naa10*-Myc and 35S::*Naa15*-Flag, were extracted and immunoprecipitated with anti-Myc or -Flag antibodies. The immunoprecipitates were separated by SDS-PAGE and immunoblotted with anti-Flag or -Myc antibodies. The antibodies for immunoprecipitation are indicated above the gels and the antibodies for immunoblotting are indicated to the right of the gels. The star indicates non-specific bands. (C) Total proteins from wild-type and complemented *naa10-1* ^*–/–*^ transgenic plants were extracted and then immunoprecipitated with anti-Myc or -Naa15 antibodies. The immunoprecipitates were separated by SDS-PAGE and immunoblotted with anti-Naa15 or -Myc antibodies.

Naa10 and Naa15 are the catalytic and auxiliary subunits of NatA, respectively, in yeast and human cells (Starheim *et al.*, 2012). Naa10 and Naa15 were localized to the cytosol (see Supplementary Fig. S3), consistent with their function as protein NATs. To determine whether they interact and function in the same complex in plants, we first examined their interaction in yeast cells. No interaction between Arabidopsis Naa10 and Naa15 was detected in a yeast two-hybrid assay (Supplementary Fig. S4), indicating either no direct interaction between the proteins or that their interaction is weak or transient. We then examined their interaction *in planta* using a co (CO)-immunoprecipitation (IP) assay. An interaction between Arabidopsis Naa10 and Naa15 was detected by IP and Co-IP in pairwise labeling and label-swapping experiments conducted in tobacco cells overexpressing Naa10 and Naa15 (Fig. 7A, B).

To examine their interaction in Arabidopsis, we generated anti-Naa15 antibodies by immunizing rabbits with purified bacterial cell-produced Naa15, and complemented *naa10* lines expressing Naa10-Myc. Their interaction was confirmed as Naa10 could immunoprecipitate Naa15 (and vice versa) in Arabidopsis (Fig. 7C). Thus, our results suggest that Naa10 and Naa15 exist in the same protein complex. This is consistent with their roles as the catalytic and auxiliary subunits of NatA and the similar phenotypes observed for *naa10* and *naa15* in Arabidopsis.

## Discussion

During early embryonic development in Arabidopsis, the precursors of all major cell types are generated; these precursors give rise to organ initials, which regulate post-embryonic patterning. Therefore, elucidating the mechanisms underlying pattern formation during early embryogenesis is critical for understanding plant development. In the present study, we examined the role of protein N-terminal acetylation in embryogenesis in Arabidopsis.

### NatA is required for asymmetric division of the hypophysis and formation of the QC in the root meristem

Naa10 and Naa15 are the catalytic and auxiliary subunits of NatA, respectively, in yeast and human cells (Starheim *et al.*, 2012); they are also conserved in the Arabidopsis genome (see Supplementary Figs S1 and S2). We found that the mutation of Naa10 caused irregular division of the hypophysis, such that no functional QC or columella stem cell was generated (Fig. 2). We also found that the distribution or/and response of auxin in early embryos and localization of PIN1 were disrupted in *naa10* embryos during embryogenesis (Figs 3 and 4). The expression of *DR5* in early globular embryos of *naa10* is strikingly similar to the expression of *DR5* in the embryos of *pin1/3/4/7* (Robert *et al.* 2013). Based on Fig. 4, it seems that the expression pattern of *PIN1* is affected, more so than the actual intracellular localization in *naa10*. If PIN1 is missing or not basally localized in the inner cells of the proembryo, the procambium is not formed accurately, which might lead to defects in the asymmetric division of the hypophysis and early embryonic patterning. However, we do not know how protein N-terminal acetylation can affect the expression of *PIN1* at this stage. Given that auxin accumulation in the hypophysis is critical for hypophysis differentiation, the aforementioned defects in auxin distribution/auxin response and PIN1 localization may lead to asymmetric division of the hypophysis (or vice versa).

Linster *et al.* (2015) showed that Naa10 and Naa15 from Arabidopsis interacted in human embryonic kidney 293 cells by expressing Naa10 and Naa15 in this human cell line. This observation was confirmed in the present study, both in tobacco and in Arabidopsis, using anti-tag and -Naa15 antibodies that were generated specifically for this purpose (Fig. 7). We also found that the mutation of Naa10 and Naa15 produced similar phenotypes in the hypophysis of Arabidopsis plants (Figs 2 and 6). These results suggest that NatA is required for asymmetric division of the hypophysis and formation of the QC in the root meristem, and that NatA must be intact to function as an NAT in Arabidopsis.

### NatA is required for the correct patterning of lower-tier cells, upper-tier cells, and the entire embryo

Once formed, a zygote undergoes asymmetric division, yielding an apical cell and a basal cell. The apical cell gives rise to the entire embryo, except for its basal end, whereas the basal cell generates the hypophysis and extra-embryonic suspensor (Jenik *et al.*, 2007). We observed that a loss of function in NatA in *naa10* and *naa15* plants led to a defect in hypophysis differentiation, and that no functional QC or columella stem cell was generated (Figs 2 and 6). However, the effect of NatA on asymmetric division of the hypophysis is not specific, as defects in other parts of the early embryo were observed in both *naa10* and *naa15* plants (Figs 2 and 6). For example, protodermal cell division was delayed, protodermal cell size was increased, and the outer portion of the embryo was irregular during the early embryonic stage in *naa10* and *naa15* plants (Figs 2 and 6). In addition, inner-cell division was delayed and irregular, while the shape and separation of the inner cells were difficult to resolve in *naa10* and *naa15* beginning at the early globular stage (Figs 2 and 6); thus, embryo development in *naa10* and *naa15* was arrested at the globular stage (Figs 2 and 6). Therefore, NatA is required for patterning of the entire embryo in Arabidopsis.

### Protein N-terminal acetylation controls embryogenesis in Arabidopsis

Patterning of the embryo is precisely controlled during embryogenesis in Arabidopsis. Much is known about the mechanisms that regulate embryo development and cell fate determination at the transcriptional level, and a number of transcriptional cascades have been revealed (Schlereth *et al.*, 2010; De Rybel *et al.*, 2013). However, little is known about the regulation of embryo patterning at the co- or post-translational level.

In the present study, we observed that a loss of function of NatA, a conserved NAT, caused defects in cell fate determination in the hypophysis and in the entire embryo (Figs 2 and 6), indicating that protein N-terminal acetylation is required for embryo pattern formation. More than 1000 proteins are N-terminal-acetylated in Arabidopsis (Bienvenut *et al.*, 2012). However, the proteins targeted by NatA and whether their N-terminal acetylation is required for embryo patterning are unknown. The targets of NatA could be proteins known to be required for embryo patterning (e.g. TOM7); however, they could also be unknown proteins. More than ten proteins required for embryogenesis in Arabidopsis are predicted targets of NatA, including ARF9, TOM7, SCR, TOAD2, AGO10, LHW, TPL, WOX5, WOX8, and WOX9 (https://bioweb.i2bc.paris-saclay.fr/terminator3/). It would be interesting to investigate whether these NatA targets mediate embryo pattern formation in Arabidopsis.

## Supplementary data

Supplementary data can be found at *JXB* online.

Table S1. Primers used for PCR.

Figure S1. Naa10 is evolutionarily conserved among eukaryotes.

Figure S2. Naa15 is evolutionarily conserved among eukaryotes.

Figure S3. Subcellular localization of Naa10 and Naa15 in *N. benthamiana* and Arabidopsis.

Figure S4. No physical interaction was detected between Naa10 and Naa15 in a yeast two-hybrid assay.

## Funding

This work was supported by grants from the National Basic Research Program (973 Program) of the Ministry of Science and Technology of China (2012CB114200 and 2012CB910900), and from the Beijing Municipal Government Science Foundation (CIT&TCD20150102).

## Author Contribution

The individual author contributions were as follows: conceptualization, LM and JF; investigation, JF, RL, JY, SM, CW, YL, and YC; writing – original draft, LM and JF; review and editing, LM and JF; funding acquisition, LM and YC; supervision, LM.

## Supplementary Material

Supplementary Data
