# Supplementary material for: Protein N-terminal acetylation is required for embryogenesis in Arabidopsis
Source: J Exp Bot. 2016 Jul 6;67(15):4779–89. doi: 10.1093/jxb/erw257 (PMC4973746; doi:10.1093/jxb/erw257)
Supplement: Supplementary Data [file supp_67_15_4779__index.html]

Protein N-terminal acetylation is required for embryogenesis in Arabidopsis — Protein N-terminal acetylation is required for embryogenesis in Arabidopsis — Supplementary Data 

# Protein N-terminal acetylation is required for embryogenesis in Arabidopsis

## Supplementary Data

Data files

- supplementary\_table\_S1\_figures\_S1\_S4.pdf - Supplementary Data
