# Supplementary material for: Protein N-terminal acetylation is required for embryogenesis in Arabidopsis
Source: J Exp Bot. 2016 Jul 6;67(15):4779–89. doi: 10.1093/jxb/erw257 (PMC4973746; doi:10.1093/jxb/erw257)
Supplement: Supplementary Data [file supp_erw257_supplementary_table_S1_figures_S1_S4.pdf]

SUPPLEMENTARY DATA

Supplemental Table S1. The primers used in this study.

naa10-1	FP	5'-TGTGTCTTCCTGAGAACTACCAG-3'
naa10-1	RP	5'-GCTGAGTTAAAGGTCACTGATTG-3'
naa10-1	LB	5;-AACGTCCGCAATGTGTTATTAAG-3'
naa15-1	FP	5'-CCTTCTACACTATTGTGGACATTG-3'
naa15-1	RP	5'-CACCTGATAGCTCTGATTGCTGC-3'
naa15-1	LB	5'-GCTTCCTATTATATCTTCCCAAATTACCAATACA-3'
naa15-3	FP	5'-CATTCCTCGTTTGCTTGTTTGTAG-3'
naa15-3	RP	5'-CGACTGATTATCATTAACGTAGAGAAC-3'
naa15-3	LB	5'-GAAATGGATAAATAGCCTTGCTTCC-3'
Naa10-Myc, *Naa10*-GFP	XbaI-FP	5'-GCTCTAGAATGGTTTGCATCAGGCGAG-3'
Naa10-Myc, *Naa10*-GFP	RP-KpnI	5'-GGGGTACCTCATTTGGAAACTGCTTTACCATC-3'
Naa10-AD, *Naa10*-BD	EcoRI-FP	5'-GGAATTCATGGTTTGCATCAGGCGAGC-3'
Naa10-AD, *Naa10*-BD	RP-SmaI	5'-TCCCCCGGGTCATTTGGAAACTGCTTTACCATCTAC-3'
Naa15-AD, *Naa15*-BD	EcoRI-FP	5'-GGAATTCATGGGGGCTTCGCTTCCTCC-3'
Naa15-AD, *Naa15*-BD	RP-SmaI	5'-TCCCCCGGGTCAGGTTGCAACTGAGAGGCTC-3'
pNaa15::Naa15-Flag, Naa15-YFP	BamHI-Naa15	5'-CGGGATCCATGGGGGCTTCGCTTCCTCC-3'
pNaa15::Naa15-Flag, Naa15-YFP	Naa15-SmaI	5'-TCCCCCGGGGGTTGCAACTGAGAGGCTCTTG-3'
pNaa15::Naa15-Flag, Naa15-YFP	SmaI-Flag	5'-TCCCCCGGGCTGCAGGAATTCGATATCAAGC-3'
pNaa15::Naa15-Flag, Naa15-YFP	Flag-SacI	5'-CGAGCTCTTAAATTCGAGCTCTGTCGACTG-3'
pNaa15::Naa15-Flag, Naa15-YFP	HindIII-pNaa15	5'-CCCAAGCTT TTCTTATTGCACTGCTTC-3'
pNaa15::Naa15-Flag, Naa15-YFP	pNaa15-BamHI	5'-CGGGATCC TTTATTGCGCTACTGAGAGAGCTC-3'

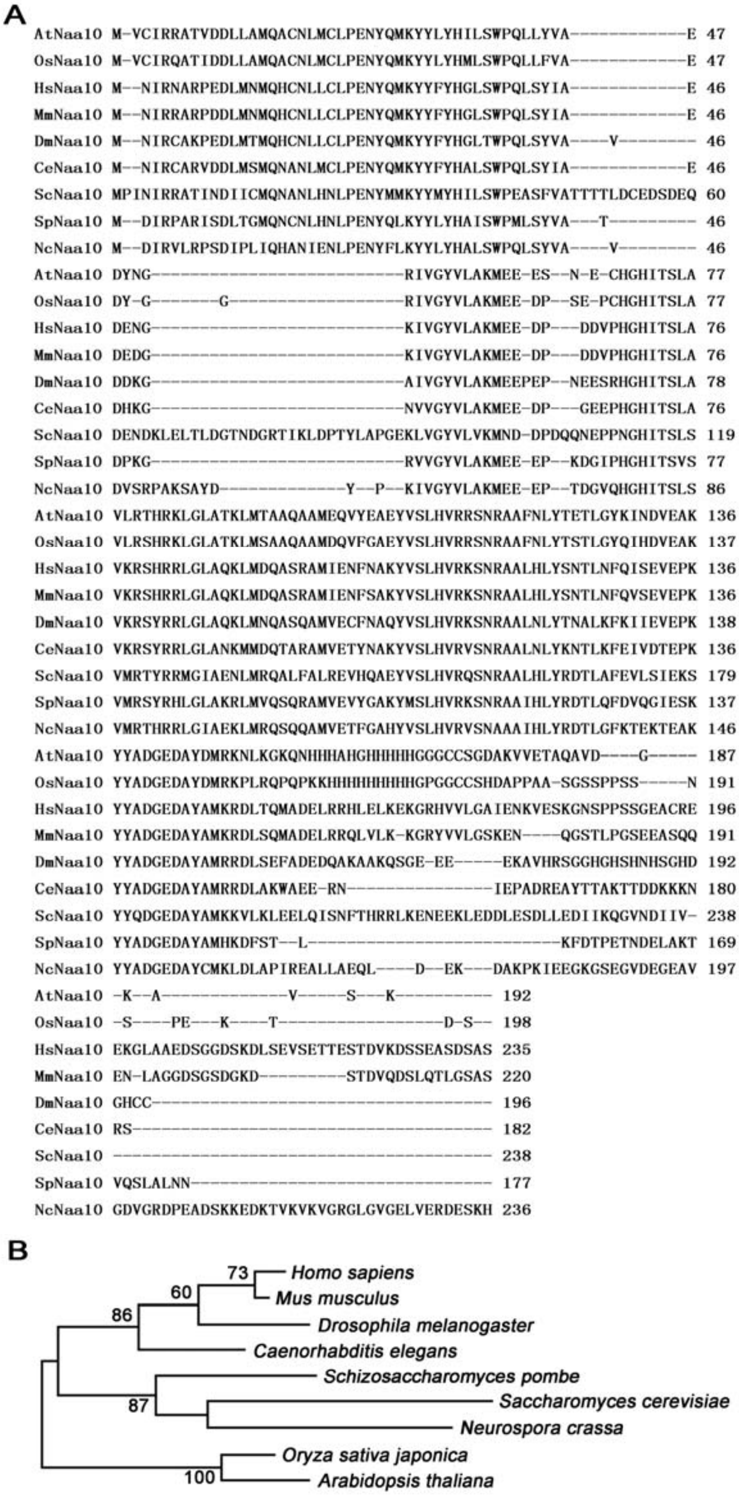

Supplemental Figure S1. Naa10 is evolutionarily conserved among eukaryotes.

(A) Amino acid sequence alignment of Naa10 orthologs from multiple taxa. The accessions of the Naa10 homologs are: *Homo sapiens*, NP_003482; *Mus musculus*, BAB27052; *Drosophila melanogaster*, NP_648378; *Caenorhabditis elegans*, NP_501392; *Oryza sativa japonica*, NP_001054011; *Arabidopsis thaliana*,

NP_196882; *Schizosaccharomyces pombe*, NP_594309; *Saccharomyces cerevisiae*, NP_011877; and *Neurospora crassaOR74A*, XP_011394752.

(B) The phylogenetic tree of the Naa10 homologs was constructed using the maximum-likelihood method with MEGA 6.

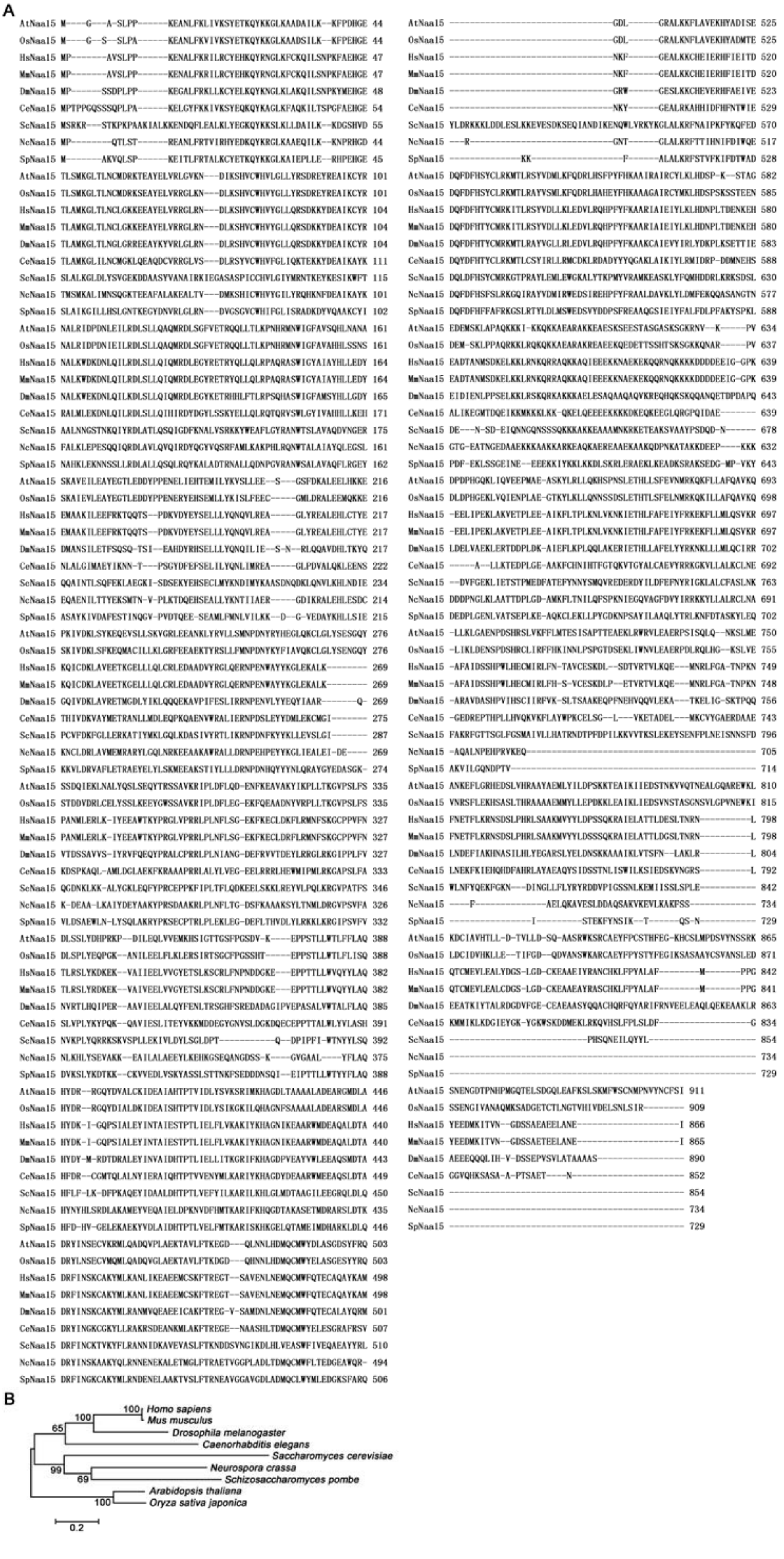
A
B
Homo sapiens
Mus musculus
Drosophila melanogaster
Caenorhabditis elegans
Saccharomyces cerevisiae
Neurospora crassa
Schizosaccharomyces pombe
Arabidopsis thaliana
Oryza sativa japonica
100
100
65
99
69
100
0.2

Supplemental Figure S2. Naa15 is evolutionarily conserved among eukaryotes.

(A) Amino acid sequence alignment of Naa10 orthologs from multiple taxa. The accessions of the Naa10 homologs are: *Homo sapiens*, NP_476516; *Mus musculus*, NP_444319; *Drosophila melanogaster*, NP_573384; *Caenorhabditis elegans*, NP_497180.3; *Oryza sativa japonica*, NP_001043582; *Arabidopsis thaliana*, NP_001185448; *Schizosaccharomyces pombe*, NP_588160; *Saccharomyces cerevisiae*, AJU80945; and *Neurospora crassaOR74A*, XP_962452.

(B) The phylogenetic tree of the Naa15 homologs was constructed using the maximum-likelihood method with MEGA 6.

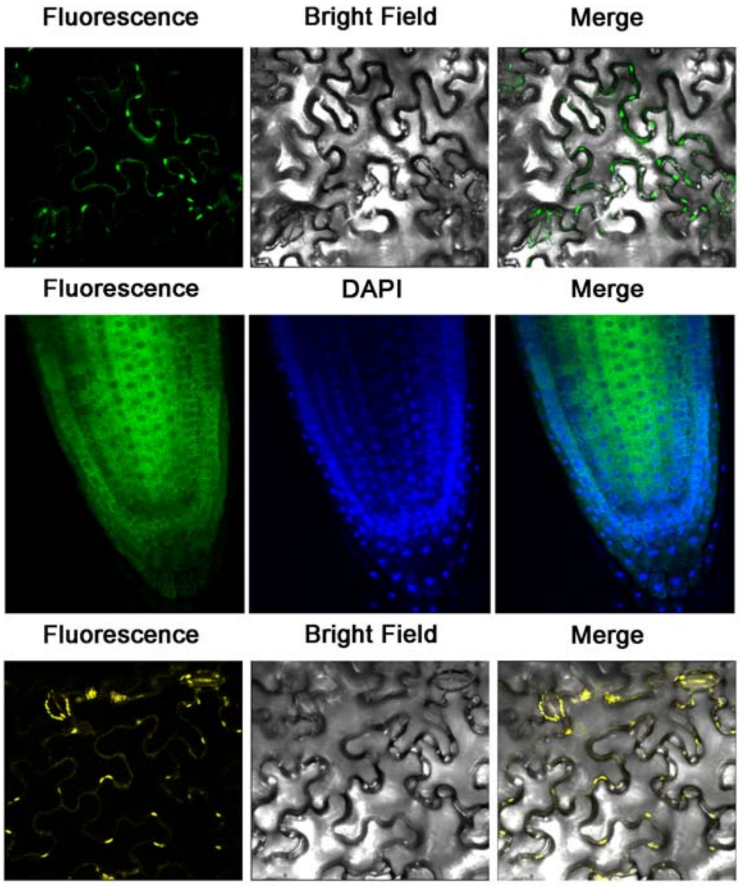

Supplemental Figure S3. Subcellular localization of Naa10 and Naa15 in *N. benthamiana* and root of transgenic *Arabidopsis*.

The *Naa10* and *Naa15* CDSs were fused to GFP and YFP, respectively, driven by the 35S promoter, and cloned into pCambia1300. Naa10-GFP or Naa15-YFP was expressed in *N. benthamiana* leaf epidermal cells (top panel and bottom panel, respectively), *p35S::Naa10-GFP* was transformed into *Arabidopsis* (middle panel). The subcellular localization of Naa10-GFP or Naa15-YFP was observed by confocal laser scanning microscopy..

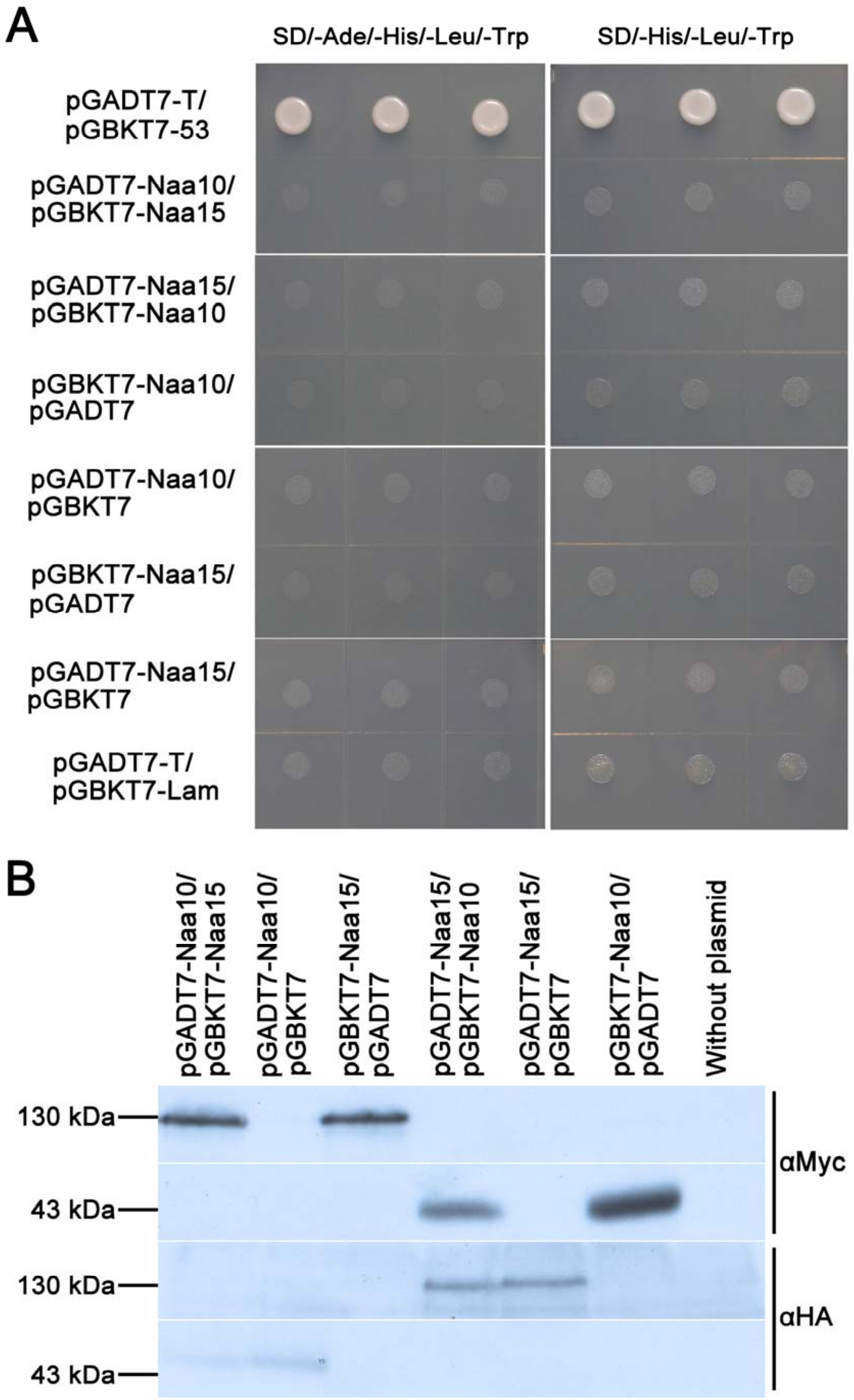

Supplemental Figure S4. No physical interaction was detected between Naa10

and Naa15 in a yeast two-hybrid assay.

(A) There was no physical interaction between Naa10 and Naa15 in yeast. Positive control: pGADT7-T/pGBKT7-53; negative control: pGADT7-T/pGBKT7-Lam. The interaction between Naa10 and Naa15 was assessed by growth on SD/-His/-Leu/-Trp and SD/-Ade/-His/-Leu/-Trp media.

(B) Both Naa10 and Naa15 were expressed in yeast. HA-specific antibodies were used to detect the proteins expressed as a fusion to an HA epitope tag from pGADT7. c-Myc-specific antibodies were used to detect the proteins expressed as a fusion with the c-Myc epitope tag from pGBKT7. AH109 cells without plasmid transformation were used as a negative control.
